# A Systematic Review of the Incidence, Risk Factors and Case Fatality Rates of Invasive Nontyphoidal *Salmonella* (iNTS) Disease in Africa (1966 to 2014)

**DOI:** 10.1371/journal.pntd.0005118

**Published:** 2017-01-05

**Authors:** Ifeanyi Valentine Uche, Calman A. MacLennan, Allan Saul

**Affiliations:** Novartis Vaccines Institute for Global Health, Siena, Italy; Oxford University Clinical Research Unit, VIET NAM

## Abstract

This study systematically reviews the literature on the occurrence, incidence and case fatality rate (CFR) of invasive nontyphoidal *Salmonella* (iNTS) disease in Africa from 1966 to 2014. Data on the burden of iNT*S* disease in Africa are sparse and generally have not been aggregated, making it difficult to describe the epidemiology that is needed to inform the development and implementation of effective prevention and control policies. This study involved a comprehensive search of PubMed and Embase databases. It documents the geographical spread of iNTS disease over time in Africa, and describes its reported incidence, risk factors and CFR. We found that Nontyphoidal *Salmonella* (NTS) have been reported as a cause of bacteraemia in 33 out of 54 African countries, spanning the five geographical regions of Africa, and especially in sub-Saharan Africa since 1966. Our review indicates that NTS have been responsible for up to 39% of community acquired blood stream infections in sub-Saharan Africa with an average CFR of 19%. *Salmonella* Typhimurium and Enteritidis are the major serovars implicated and together have been responsible for 91%% of the cases of iNTS disease, (where serotype was determined), reported in Africa. The study confirms that iNTS disease is more prevalent amongst Human Immunodeficiency Virus (HIV)-infected individuals, infants, and young children with malaria, anaemia and malnutrition. In conclusion, iNTS disease is a substantial cause of community-acquired bacteraemia in Africa. Given the high morbidity and mortality of iNTS disease in Africa, it is important to develop effective prevention and control strategies including vaccination.

## Author Summary

Although *Salmonella* are a common global cause of mild gastro-intestinal illness that usually presents with self-limiting diarrhoea, the invasive nontyphoidal form of *Salmonella* disease manifests as bacteraemia which often presents only with fever. If left untreated, iNTS disease often results in death. iNTS disease is more common amongst people with impaired immunity, and in developing countries particularly in Africa. Data on the epidemiology of iNTS disease in Africa are sparse, making it difficult to estimate the true disease burden. This study aggregates all published cases of iNTS disease in Africa from 1 Jan 1966 up to 31 Dec 2014 and shows the relative importance of NTS as a cause of community acquired bacteraemia and deaths in Africa. The findings are important in raising awareness of iNTS disease and driving research to help develop effective control and preventive strategies to limit the disease. Important interventions needed for iNTS disease include rapid diagnostic tests and vaccines, as well as measures to improve hygiene.

## Introduction

Nontyphoidal *Salmonellae* (NTS) are a major cause of food borne infections throughout the developed and developing world [1]. Although infection most often results in self-limited acute gastroenteritis, NTS have been identified as a major cause of invasive bacterial infections in infants and young children in sub-Saharan Africa and HIV-infected individuals of all ages [2,3]. Invasive NTS (iNTS) disease is recognized as a problem in developed countries in young infants, the elderly and immunocompromised [4]. iNTS disease is caused mainly by *Salmonella enterica* serovars Typhimurium and Enteritidis [5,6].

NTS gastroenteritis is generally understood to be acquired from animal reservoirs, unlike *Salmonella* Typhi and *Salmonella* Paratyphi, where the only recognized reservoir is man. Transmission of gastroenteritis-causing NTS to humans can occur by many routes, including consumption of animal food products, especially eggs, poultry, undercooked meat, produce contaminated with animal waste, contact with animals or their environment, and contaminated water [7,8].The African strains responsible for iNTS disease are characterized by genome degradation and appear to be increasingly adapted to an invasive lifestyle [9]. The relative role of animal reservoirs and human to human transmission of strains causing iNTS disease is unclear [10,11]. iNTS disease is diagnosed definitively by blood or bone marrow culture, usually with low sensitivity [12]. It is also impossible to diagnose using clinical symptoms alone due to the lack of pathognomonic features [5,6]. There is currently no commercially-available rapid diagnostic test for iNTS disease.

Bacteraemia is an important cause of severe and often fatal disease globally, especially in developing countries (where it substantially contributes to childhood deaths) [2,13]. Invasive forms of *Salmonella* disease include enteric fevers (typhoid and paratyphoid fevers) and NTS bacteraemia and are important causes of morbidity and mortality in Asia and Africa [3,14,15]. In a review article of bacteraemia in Africa from 2010, NTS was found to be responsible for 29.1% of all bloodstream infections [16]. Nevertheless, the data on NTS bacteraemia in Africa are limited with no current aggregate data, so it has been difficult to estimate the burden of NTS bacteraemia in Africa. Such information is vital for planning and implementing cost-effective solutions [17] to tackle iNTS disease, especially in Africa with its fragile health-care systems. Infectious diseases are a major obstacle to human development in the African region with people suffering from an extensive range of potentially preventable and treatable conditions including invasive *Salmonella* disease. Adequate evidence on the burden of *Salmonella* infections would enable health policy makers to make informed decisions on the need for vaccines against *Salmonella* infections in their country and region.

The aim of the present study is to address this knowledge gap by conducting a review of the available reports on NTS bacteraemia in Africa and to describe the epidemiology of the disease; in order to inform effective prevention and control strategies, including vaccination. Specifically, the study describes all the geographical locations in Africa that have reported iNTS disease cases, and determines incidence and proportion of bacteraemia caused by NTS (by whole population and patient groups), together with risk factors and CFR of NTS bacteraemia.

## Methods

### Search strategy

We searched PubMed (including MEDLINE) and Embase. The search string used was: (algeria OR angola OR benin OR botswana OR “burkina faso” OR burundi OR cameroon OR “cape verde” OR “central african republic” OR chad OR comoros OR “ivory coast” OR “cote d ivoire” OR congo OR djibouti OR egypt OR “equatorial guinea” OR eritrea OR ethiopia OR gabon OR gambia OR ghana OR guinea OR “guinea bissau” OR kenya OR lesotho OR liberia OR libya OR madagascar OR malawi OR mali OR mauritania OR mauritius OR morocco OR mozambique OR namibia OR niger OR nigeria OR rhodesia OR rwanda OR “sao tome” OR senegal OR seychelles OR “sierra leone” OR somalia OR “south africa” OR sudan OR swaziland OR tanzania OR togo OR tunisia OR uganda OR “western sahara” OR zambia OR zimbabwe OR Africa) AND (fever OR fevers OR bacteremia OR bacteremias OR bacteremic OR bacteraemia OR bacteraemias OR bacteraemic OR septicemia OR septicemias OR septicemic OR septicaemia OR septicaemias OR septicaemic OR salmonella OR salmonellas OR salmonellae OR “blood stream infection” OR “blood stream infections” OR “blood stream pathogen” OR “blood stream pathogens” OR febrile) AND (infant* OR child* OR adolescent* OR adult* OR patient OR patients OR human OR travel* OR communit* OR village* OR participant* OR volunteer* OR subject* OR incidence OR hospital OR man).

We used the United Nations list of 54 African Sovereign states as the basis for searching, modified by including Rhodesia (name had changed during the search period) and both the English and French names for Ivory Coast. Initial tests showed that “Congo” found all references to the Democratic Republic of the Congo as well as Republic of the Congo. Similarly, “Sudan” found both Sudan and South Sudan. Although it is not recognized by the United Nations as a sovereign state, we also included “Western Sahara” in case there were reports from this area.

PubMed and Embase have, an English translation for all paper titles regardless of language, English key words (i.e. MESH terms in PubMed) and usually an English Abstract. These were the only fields searched in the initial searching for papers of any language and assumes that the English terms used in the search would be sufficient to identify papers in any language for the first pass.

Initial searches failed to find any papers prior to 1966, search results were limited to publications from 1^st^ Jan 1966 up to 31^st^ December, 2014. We imported the full texts/abstracts of the search result into Quosa Information Manager software (Quosa) [18] and a full text search was done through Quosa for articles containing the term “Salmonell*”.

To ensure a comprehensive search of the literature, especially for reports within the last five years, we did an independent search on Embase database using a similar search string and strategy as above (with limit publication dates from 2009 to 2014). Additional reports that were not retrieved from the PubMed search were obtained and reviewed for inclusion.

### Selection criteria

The full text of the search results of online articles/abstracts were reviewed independently by the study authors (IVU, CAM and AS) with the aim of including articles that used blood culture to isolate NTS from humans in Africa Inconsistences between the papers included by the different authors were resolved by consensus. The full text version either obtained on line or ordered, when only the abstract was available on line, of potentially relevant articles was retrieved and reviewed critically using predetermined inclusion and exclusion criteria for the study. This was done for articles, regardless of the published language. The reference sections of retrieved full text articles were reviewed critically in search of further potential articles for inclusion.

#### Inclusion criteria

Studies were included if they:

1. Reported NTS isolated by blood culture
2. Were conducted in and recruited subjects from Africa;

#### Exclusion criteria

Studies were excluded if they:

1. Described NTS disease isolated by stool culture only without blood culture result
2. Did not specify serovar of *Salmonella enterica* isolated (for example, *Salmonella* Typhi or one of the serotypes causing NTS)

#### Validity assessment

Study validity was established by use of the selection criteria described above, thereby excluding studies that were thought likely to have results that are either inaccurate, not representative of the reported population or could otherwise not be compared with studies included in the analysis. Studies were not excluded on the basis of potential variability of microbiological techniques and identification of culture isolates.

### Review of the selected literature

#### Data extraction and collection process

Relevant descriptive and quantitative variables were extracted from each of the selected article. A standardised template was used for the data extraction in the form of a Microsoft Excel 2013 workbook with each column of the database corresponding to one of the fields in the template. Double data extraction and entry was performed to ensure accuracy. The variables extracted from each article included name of journal, title of article, publication date, study location (including city, country and region in Africa), study period, patient age, and primary eligibility criteria). Quantitative data collected included number of potential study participants, subjects enrolled, participants who had blood cultured, significant pathogens isolated, NTS pathogens isolated; iNTS disease incidence (if stated), CFR of iNTS disease, and proportion of study participants with risk factors such as infection with malaria or human immunodeficiency virus (HIV) (if known). Where publications separately included data derived from more than one cohort of subjects (e.g. different age groups), each unique study was included as a separate entry in the table. The fields used are listed in S1 Table.

#### Data analysis

Data were cleaned and a descriptive analysis performed with the aid of Microsoft Excel 2013. All reported measures of disease frequency were directly drawn from the literature.

Duplicate reports of iNTS disease cases were sorted and removed from the final analysis or, where this was not possible, clearly identified as potentially overlapping (See supplementary S2 Table for listing of relevant reports). The sorting was done by grouping all reports according to country and then comparing variables including study dates, name and location of the study site, age group of subjects reported, number of blood cultures taken and number of NTS isolated across the studies from each country for potential overlaps.

Incidence data were extracted directly from studies where this was stated and summarized according to sub-group classification, subjects, location, study date, groups at risk and possible risk factors. Incidence was derived in a few studies by dividing the total number of NTS isolated by the population at risk per unit time (year) and stated as cases per 100,000 person year of observation or per 100,000 population per year.

Proportion of NTS as the cause of community acquired blood stream infection was calculated using the formula: *number of* NTS *bacteraemic cases divided by total number of significant/pathogenic bacteraemia in the relevant studies*. Relevant studies included prospective and retrospective hospital-based blood culture series for subjects presenting with fever with no known focus of infection or groups of subjects not selected for possible associated risk factors as anaemia, HIV, malaria and malnutrition.

CFR data were extracted directly from the studies where given. CFR was calculated using the formula: *death amongst* NTS *bacteraemia cases divided by total* iNTS *disease cases multiplied by 100*. The CFR data were then summarized according to subjects, location, study period and possible risk factors.

Summary data for risk factors associated with iNTS disease in Africa were extracted directly from the studies where available. Statistically-significant measures of association, such as odds ratio (OR), risk ratio (RR) and prevalence proportion were extracted from the studies.

## Results

### Search results

**Fig 1:**
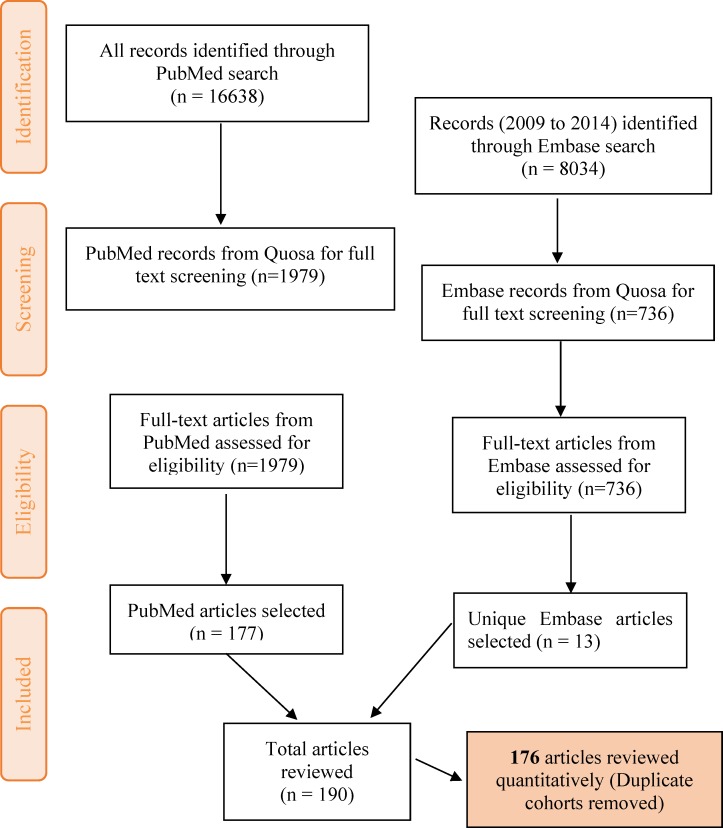
Strategy for selection of eligible articles. Adapted from the PRISMA group 2009 flow diagram [203].

The online database search performed on PubMed (completed in February, 2015), using the search string and limiting the results from 1^st^ January 1966 to 31^st^ December 2014, yielded 16,638 articles. These were retrieved in Quosa and a full text search for the term ‘*Salmonella*’ using Quosa yielded a subset of 1,979 articles. The abstract and full text (where available) of the 1,979 articles obtained were reviewed manually for relevance based on our criteria. 177 articles were finally selected from PubMed and entered into the database (Fig 1). We found more publications using our search string with all the countries in Africa included by name, than with “Africa” alone, and by not restricting publications by using ‘human’ as a search term. The articles retrieved in Quosa were published in 16 languages of which French (216 articles) was the most common following English.

A similar search strategy was used on the Embase database with the initial search results limited to articles in Embase only, humans and the period 2009 to 2014, yielding 8034 articles (in February, 2015). 736 articles were obtained following a full text search for the term ‘*Salmonella*’ in Quosa. Thereafter, the full texts of these articles were reviewed manually and 13 additional unique articles not obtained from the initial PubMed search were added to the database (including 9 abstract-only articles).

One hundred and ninety articles [2,3,10,11,13,19–202] were obtained following the literature search and used for the descriptive analysis in our review (Fig 1). Following a further review of these articles, 14 (S2 Table) reporting cases or cohorts of iNTS disease already reported in another published article were excluded from the quantitative analysis, leaving 176 articles including 12 studies with possibly overlapping data (S2 Table). These 176 studies included 223 distinct subject cohorts.

The reports varied in their methodology. Three of the 176 articles were reports from ill subjects in a longitudinal, community-based surveillance. 159/176 (90%) of all articles retrieved are reports of isolates obtained from studies conducted on ill subjects presenting to a health facility setting (hospital or clinic). In fourteen of the reports, we could not determine the original basis for selecting the subjects (E.g., retrospective analysis of microbiology laboratory samples, follow up analyses of bacteraemia in patients previously selected for another study). However, most of these 14 were probably from ill subjects presenting to a health facility.

Only 9 (5%) were designed to derive an estimate of the population-based incidence of iNTS disease in Africa; 76 (43.2%) were prospective hospital-based studies with patients recruited during the course of the study; 22 (12.5%) were retrospective studies with analysis of existing hospital or laboratory records; and the remaining 69 (39.2%) are case reports, series, conference abstracts or outbreak reports.

A total of 18,931 isolates of NTS were reported in this review. These included 9,084 isolates of *Salmonella enterica* serovar Typhimurium (*S*. Typhimurium) (48%), 2,801 isolates of *S*. Enteritidis (15%), 1,215 other serovars (6%) and 5,831 ‘not further typed’ (31%).The category ‘other serovars’ includes, but is not limited to, less common serovars: 197 Infantis, 155 Wien, 88 Dublin, 71 Newport, 71 Bovis-morbificans, 92 Isangi, 24 Heidelberg, 8 Havana and 9 Ordonez [11,40,49,65,68,71,91,104,108]. *Salmonella* Typhimurium is therefore the most common serovar reported for iNTS disease in Africa and approximately three times (48% vs 15%) more common than *S*. Enteritidis.

### Geographical location of sites reporting iNTS disease in Africa

**Fig 2:**
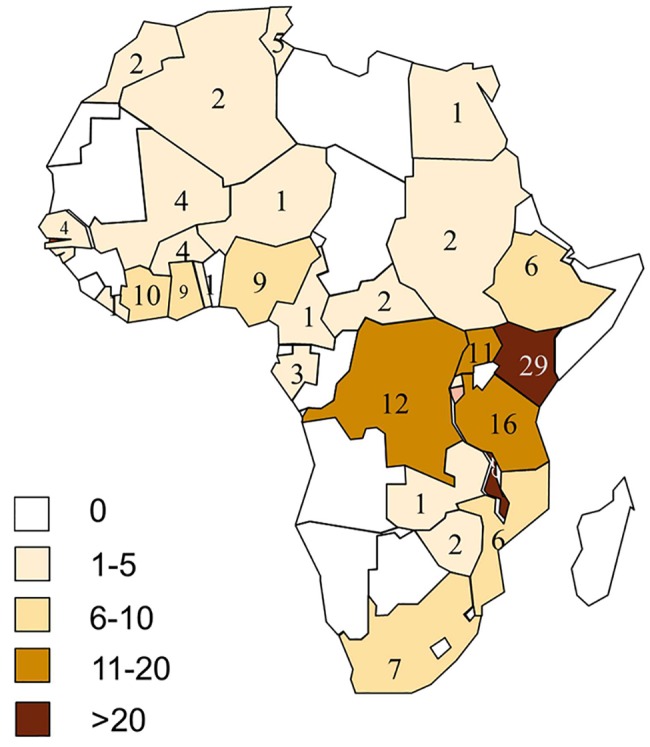
Map of Africa showing number of publications from countries reporting NTS blood culture isolates. Regions are indicated using coloured boundaries based on United Nations classification

iNTS disease was reported in 33 countries across the five geographical regions of Africa based on the United Nations classification (Fig 2). The majority, 53% (94/176) of the reports were from the Eastern Africa region (including 28 reports from Kenya, 24 from Malawi, 16 from Tanzania and 10 from Uganda); 26% (46/176) from the Western Africa region (including 9 reports from Cote d’Ivoire, 9 from Nigeria and 8 from Ghana); 10% (17/176) from Central Africa (including 11 reports from Democratic Republic of Congo, 3 from Gabon and 2 from Central African Republic); 7% (12/176) from Northern Africa (including 5 reports from Tunisia and 2 each from Morocco and Algeria); and 4% (7/176)from Southern Africa (South Africa) (S3 Table).

Details of the study population or study site setting could be extracted from only 113 of the 176 reports obtained. 50.4% (57/113) of the reports were from urban sites, 38.9% (44/113) from rural sites and 10.6% (12/113) from both urban and rural sites (S4 Table).

**Fig 3:**
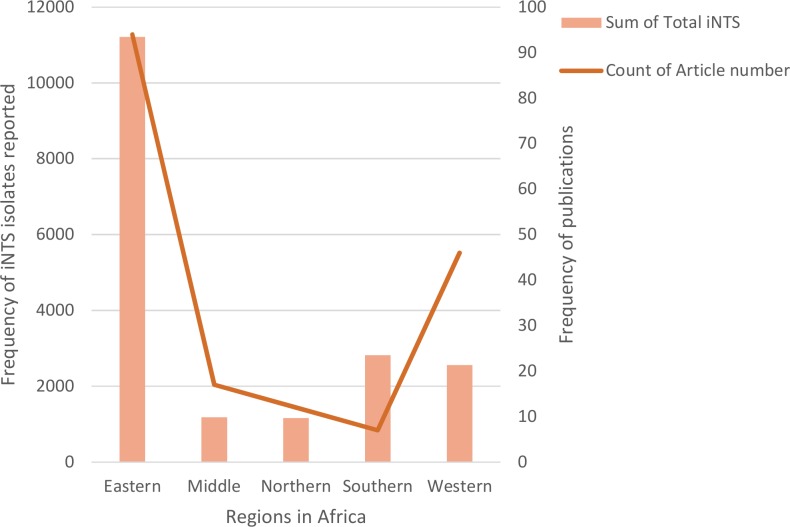
Regional distribution of iNTS disease cases reported from Africa (1966 to 2014). Graph shows total numbers of NTS isolates reported and numbers of publications in which cases were reported.

59.2% (11,211/18,931) of the total NTS isolates were reported from the Eastern Africa region (6,057 isolates from Malawi, 2,976 isolates from Kenya and 767 isolates from Uganda). 14.9% (2,819/18,931) and 13.5% (2,558/18,931) of isolates were from Southern (2,819 isolates from South Africa) and Western Africa (788 isolates from Mali, 371 from Cote d’Ivoire and 345 from Ghana) respectively. 6.2% (1,180/18,931) and 6.1% (1,163/18,931) of isolates were from Central (1,003 isolates from Democratic Republic of Congo, 138 from Gabon and 34 from Central African Republic) and Northern Africa (1,023 isolates from Algeria, 100 from Tunisia and 36 from Morocco) respectively (Fig 3).

### Reports based on year of publication

**Fig 4:**
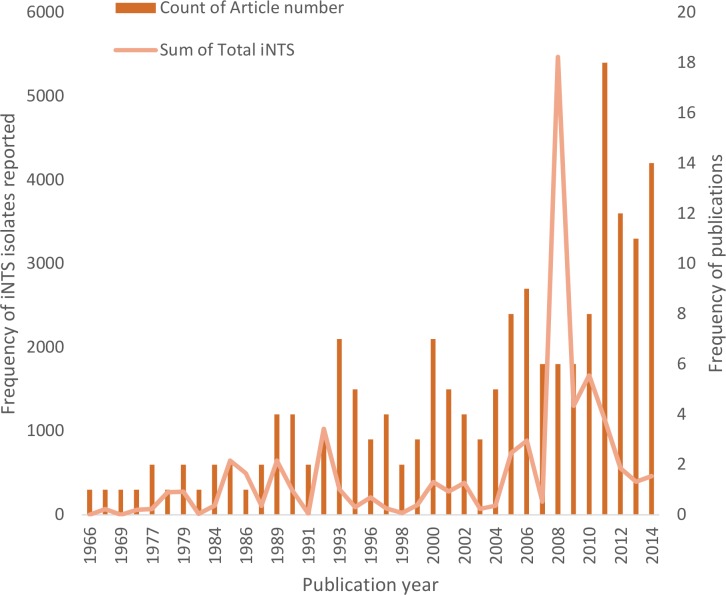
Published reports of iNTS disease in Africa by year of publication

The earliest report of iNTS disease was published in 1966 [69] and the numbers of reports of iNTS disease increased with time to a peak of 18 reports in 2011 (Fig 4). 55.7% (98/176) of the reports obtained were published within the last decade (2005 to 2014), with 35.8% (63/176) were published in the last 5 years (2010 to 2014).

A similar trend can also be observed in the total number of NTS blood culture isolates with time. 67.5% (12770/18931) of the total isolates were reported within the last ten years (2005 to 2014), while 22.3% (4218/18931) were reported in the last 5 years (2010 to 2014).

### Incidence of iNTS disease in Africa

Fourteen reports on the incidence of iNTS disease in Africa were obtained from eight countries–the Gambia, Ghana, Kenya, Malawi, Mozambique, South Africa, Tanzania & Uganda, spanning three regions, Eastern (ten studies), Southern (one) and Western (three) (Table 1). The incidence data were obtained using different methods of estimation and were from different population groups including specific age groups, HIV-infected patients and subjects with sickle cell disease. Overall, the estimated incidence of iNTS disease ranged from 1.4 per 100,000 population/year (in South African individuals of all ages in 2003 to 2004) to 2,520 per 100,000 population per year in children < 5 years of age from Ashanti, rural Ghana, in 2007 to 2009.

**Table 1:** Published reports of incidence of iNTS disease in Africa (1966 to 2014)

Region	Ref no.	Study	Study place & time	iNTS Incidence/100,000 person years (C.I)	iNTS incidence in Sub group	Subjects	Study type	Age	Total Blood Cultures	Total bacter-aemia	Population	All iNTS	Comments
Eastern	[2]	Berkley et al, 2005	Kilifi, rural Kenya; August 1998 to July 2002		<1 yr old: 170; <2 yr old: 175; <5 yr old: 88	All acute medical admissions	Hospital based, Prospective consecutive	<13 years	16,570	1,094	189,148 (15% births in study hospital)	166	Estimated minimal incidence
	[122]	Mandomando et al, 2009	Manhiça, rural Mozambique; May 2001 to April 2006	**S. Typhimurium: 78.6 (65.4–94.3);**	<1 year: 240.4 (169.1–341.8); 1–4 years: 176.2 (141.5–219.3);≥ 5 years: 4.5 (1.7–12.1)	Febrile admissions	Prospective consecutive	<15 years	19,896	1,550	140,000	263	Minimal incidence
		Mandomando et al, 2009	Manhiça, rural Mozambique; May 2001 to April 2006	**S. Enteritidis: 28.9 (21.2–38.8)**	<1 year: 108.6 (64.3–183.3); 1–4 years: 59.5 (40.8–86.7); ≥ 5 years: 1.1 (0.16–8.1)	Febrile admissions	Prospective consecutive	<15 years	19,896	1,550	140,000	101	Minimal incidence
	[129]	Mtove et al, 2011	Muheza, rural Tanzania; June 2006 to May 2010	**82 (No Confidence interval)**	2006–7: 82; 2007–8: 14; 2008–9: 17; 2009–10: 7	Severely ill admissions	Hospital based prospective	2 months to < 15 years	6,836	684	123,613 (average)	232	Minimal incidence, pooled analysis
	[131]	Muyanja et al, 2011	Entebbe, semi-urban Uganda; January 2000 to December 2008		Pre-*ART1: 730 (290–1,830); Pre-ART2: 2,930 (1,910–4490); Interim: 1100 (600–2,020); ART1: 710 (370–1,360); ART2: 800 (100–540)	HIV-1 infected outpatients	Prospective cohort	≥15 years		246	2,540	66	Periods (year) include: Pre-ART1 (2000–1); Pre-ART2 (2001–3); Interim (2003–5); ART1 (2005–7); ART2 (2007–8)
	[160]	Sigauque et al, 2009	Manhiça, rural Mozambique; May 2001 to April 2006	**120 (103–139)**	<1 year: 388 (294–512); 1-<5 years: 262 (219–314); >5 years: 6 (3–15)	Admissions with fever or malnutrition	Hospital based	<15 years	19,896	1,550	140,000 (43% children <15 years)	397	Minimal incidence
	[164]	Tabu et al, 2012	Asembo, rural Kenya; October 2006 to September 2009	**580 (229–934)**	0–4 years: 2,085 (1,181–2990); 5–9 years: 389 (106–672); 10–17 years: 24 (0–62); 18–49 years: 367 (186–550); >50 years: 232 (112–351)	Febrile or Acute respiratory illness	Prospective cohort	≤50 years	3,578	155	25,000	60	Extrapolated for patient samples not cultured or who visited another clinic (sampling)
		Tabu et al, 2012	Kibera, urban Kenya; March 2007 to February 2009	**57 (0–122)**	0–4 years: 260 (102–419); 5–9 years: 37 (1–73); 10–17 years: 0 (NA); 18–49 years: 11.2 (0–31); >50 years: 0 (NA)	Febrile or Acute respiratory illness	Prospective cohort	≤50 years	2,138	230	30,000	7	age stratified years (1–4; 5–9; 10–17; 18–49; >50) S. Typhi more common
	[174]	Williams et al, 2009	Kilifi, rural Kenya; August 1998 to March 2008	**1,580 (1,080–2,230)**	0–11 months: 360 (70–1050); 12–23 months: 1,080 (220–3,150);2–13 years: 1,430 (760–2,440)	All admissions with sickle cell anaemia	Case-control, retrospective	<14 years		108	100,000	19	Incidence in patients with Sickle cell anaemia
	[123]	Mayanja et al, 2010	Southwest Uganda; January 1996 to December 2007	**855 (617–1223)**	13–24 yrs: 713 (328–1850);	Fever without detectable malaria	Population-based HIV clinical cohort	All ages	703	159	20,000	42	37.7% HIV prevalence in cohort
					25–34 yrs: 883 (533–15720);								
					35–44 yrs: 1395 (789–2708);								
					45+ yrs: 375 (142–1332).								
					HIV negative: 37 (5–266);								
					HIV, no ART:2070(1480–2980);								
					HIV pos, ART:739 (237–3533)								
	[76]	Feikin et al, 2013	Asembo, Kenya; March 2007 to February 2010	**1200 (570–1800)**		Patients with severe acute respiratory illness	Hospital-based, prospective	< 5 years old	747	24		14	
	[83]	Gordon et al, 2008	Blantyre, Malawi; Jan 1998 to Dec 2004	**164**		Febrile, clinical sepsis	Hospital based, prospective	All ages	62,878	10,628	502053	4955	Minimal incidence–derived from the data
Southern	[11]	Feasey et al, 2010	South Africa; January 2003 to December 2004	**1.4**		All	Active laboratory surveillance data	All ages	ND	ND	46,000,000	1318	Population based; Derived from data
Western	[67]	Enwere et al, 2006	Basse & Bansang, rural Gambia; August 2000 to April 2004	**Group A- 262 (190–354)**	2–5 months: 227 (74–530); 6–11 months: 407 (237–652); 12–17 months: 238 (114–438); 18–23 months: 233 (100–458); 24–29 months: 126 (26–369)	Vaccine trial participants suspected with bacteraemia	Part of vaccine trial	2 to 29 months	7369	295	ND	92	Prospective cohort from the population
		Enwere et al, 2006	Basse & Bansang, rural Gambia; August 2000 to April 2004	**Group B- 300 (222–397)**	2–5 mo: 408 (187–775); 6–11 mo: 360 (201–594); 12–17 mo: 334 (183–561); 18–23 mo: 293 (140–539); 24–29 mo: 42 (1–236)	Vaccine trial participants	Part of vaccine trial	2 to 29 months	7369	295	ND	93	Prospective cohort from the population
	[184]	Marks et al, 2012	Agogo, rural Ghana; Jan 2010 to Oct 2011		<5 years: >600	Febrile in & outpatients	2 year Surveillance data	All ages	5134	389	38882	ND	Abstract only, survey used to adjust incidence calculation
	[137]	Nielsen et al, 2012	Ashanti, rural Ghana; September 2007 to July 2009	**2520 (2110–2940)**	Nil	Admissions	Prospective consecutive	< 5 years	1196	238	149500 (15% aged <5 years)	129	Incidence adjusted based on health-seeking behaviour

*ART anti-retroviral therapy

The incidence rates were much higher in Eastern and Western Africa compared to Southern Africa. Across the studies, the estimated incidence rates were higher amongst HIV-infected subjects [123,131], subjects with sickle cell disease[174], young children[122,160,164] and in a rural setting compared to an urban setting [164].

Three studies estimated iNTS disease in the community. These studies were conducted on subjects from all age groups, and without bias for risk factors such as HIV infection, malaria and anaemia. Estimates obtained were 1.4, 164 and >600 per 100,000 population per year from South Africa, Malawi and Ghana respectively [11,83,184].

### Proportion of NTS as a cause of community acquired blood stream infection in Africa

Fifty six studies, describing a total of 114,634 blood cultures (S5 Table) from four regions were eligible for analysis: hospital-based studies from Africa investigating the organisms causing bacteraemia in the community and not in any specific risk group. Eligible studies were from four of the five regions (all except the Northern region) and sixteen countries in Africa. NTS proportions varied between and within regions. Regional averages ranged from 8% in Southern Africa to 38% in Central Africa with an overall average of 25%. In Eastern Africa, with an average of 27%, the range was 9% in Ethiopia to 39% in Malawi while in Western Africa, with an average of 18%, the range was 8% in Nigeria to 34% in Burkina Faso.

**Fig 5:**
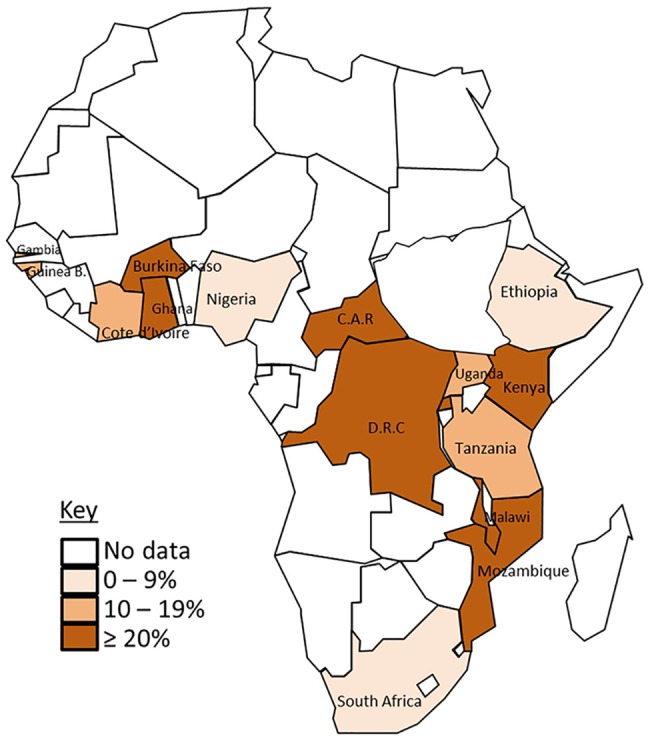
Proportion of Community acquired blood stream infections caused by NTS in African countries (1966 to 2014)

Across countries, NTS was a cause of about 8% (lowest value) of community acquired bacteraemia in both Nigeria and South Africa to a peak value of 45% in one study from Central African Republic involving 131 blood cultures (Fig 5). Variations in NTS proportion within country were found in Malawi, Kenya, Tanzania and Uganda. This suggests that it might be difficult to directly extrapolate the burden of iNTS disease within a country as well as from one country to another.

**Fig 6:**
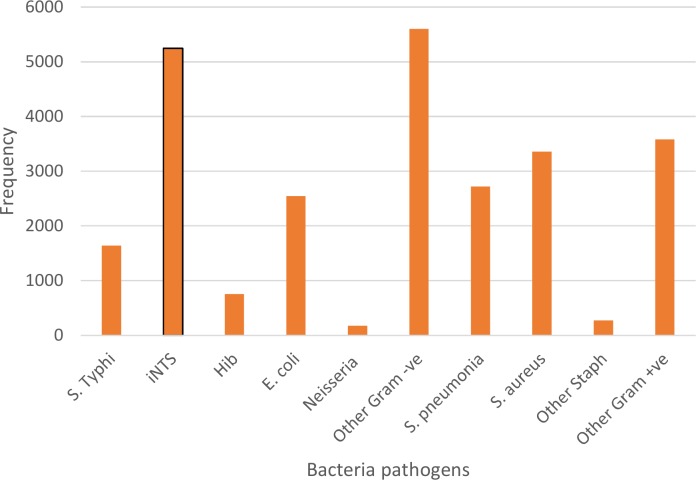
Pathogens reported from community acquired bacteraemia cases in Africa (1966 to 2014)

An analysis of the relative frequencies of different organisms causing community acquired bacteraemia in Africa was performed and it showed that NTS, *Staphylococcus aureus* and *Streptococcus pneumoniae* are most prevalent organisms isolated over time in Africa (Fig 6). Since the selection of articles included in this review excluded blood culture series without NTS, there is an element of bias favouring NTS compared to other organisms. However, the review of Reddy et al [16] also indicated the importance of iNTS as a cause of community acquired blood stream infection amongst adults and children in Africa [16].

### Risk factors associated with iNTS disease in Africa

The risk factors known to be associated with iNTS disease in Africa include HIV infection, malnutrition, malaria, young age, anaemia and rural setting (Table 2).

**Table 2:** Risk factors associated with iNTS disease in Africa (1966 to 2014)

	Risk factor	Ref no	Author, Year	Measure of association reported (Confidence interval)	Comments
1	**HIV infection**				
		[31]	Archibald et al, 2000	OR: 4.4 (0.6–93)*	P value not significant
		[2]	Berkley et al, 2005	OR: 3.21 (1.95–5.28)	OR adjusted for age, HIV infection and malnutrition
		[55]	Bronzan et al, 2007	OR: 11.6 (2.1–63.7)	Children with severe malarial anaemia
				Adjusted OR: 22.1 (3.3–146)	
		[156]	Seydi et al, 2005	Prevalence: 81% vs 36%	P = 0.00001, iNTS in patients with vs without HIV
		[81]	Gilks et al, 1990	OR: 48.2 (13–176)	S. Typhimurium only
		[51]	Blomberg et al, 2007	Prevalence: 25% vs 8.4%	P = 0.022; iNTS in HIV infected vs uninfected patients
		[151]	Peters et al, 2004	OR: 5.3 (1.2–2.2)	
2	**Malnutrition**				
		[2]	Berkley et al, 2005	OR: 1.68 (1.15–2.44)	OR adjusted for age, HIV infection and malnutrition
	Severe (WAZ<-3)	[122]	Mandomando et al, 2009	OR: 1.44 (1.08–1.91)	P = 0.004; iNTS vs Other bacteraemia; Multivariate analysis
	wasting	[137]	Nielsen et al, 2012	OR: 1.9 (1.2–3.4)	
		[160]	Sigauque et al, 2009	OR: 2.42 (1.92–3.04)	Unadjusted analysis
	Severe acute	[192]	Biggs et al, 2014	OR: 2.01 (1.15–3.52)	P = 0.014; Multivariate analysis
3	**Malaria**				
	*P*. *falciparum*	[122]	Mandomando et al, 2009	OR: 1.61 (0.91–1.47)	P = 0.8; iNTS vs Other bacteraemia; Univariate analysis
	*P*. *falciparum* in children >6 mo old	[86]	Graham et al, 2000	RR: 1.5 (1.2–2.2)	P<0.01; compared to other pathogens combined
	Recent malaria	[192]	Biggs et al, 2014	OR: 4.13 (2.66–6.44)	P<0.001; Multivariate analysis
4	**Age**				
	12–23 months vs >5 years old	[2]	Berkley et al, 2005	Prevalence: 23% vs 7%	P value (<0.001)
	≥2 vs <2 years	[55]	Bronzan et al, 2007	OR: 4.3 (0.8–22.9)	Children with severe malarial anaemia
				Adjusted OR: 9.6 (1.4–64.8)	
	2–11 months	[122]	Mandomando et al, 2009	OR: 2.07 (1.07–4.01)	P = <0.001; iNTS vs Other bacteraemia; Multivariate analysis
	1–4 yrs	[122]	Mandomando et al, 2009	OR: 3.90 (2.06–7.40)	P = <0.001; iNTS vs Other bacteraemia; Multivariate analysis
5	**Anaemia**				
	Moderate	[122]	Mandomando et al, 2009	OR: 1.86 (1.34–2.58)	P = <0.001; iNTS vs Other bacteraemia; Multivariate analysis
	Severe	[122]	Mandomando et al, 2009	OR: 3.48 (2.03–5.95)	P = <0.001; iNTS vs Other bacteraemia; Multivariate analysis
	Severe	[86]	Graham et al, 2000	Risk Ratio: 7.2 (3.4–15.3)	P<0.0001; compared to other pathogens combined
	Sickle cell	[174]	Williams et al, 2009	OR: 35.6 (16.4–76.8)	Age adjusted (<14years)
	Anaemia (Hb<7)	[151]	Peters et al, 2004	OR: 2.2 (1.1–4.5)	
	Severe	[192]	Biggs et al, 2014	OR: 2.19 (1.48–3.23)	P<0.001; Multivariate analysis
6	**Setting**				
	Rural ward residence	[192]	Biggs et al, 2014	OR: 2.23 (1.25–3.96)	P = 0.006; Univariate analysis

*OR Odds Ratio

Seven studies from Africa showed a positive association between HIV and iNTS disease in Africa. The estimated odds ratio (OR) from the studies for HIV-infected individuals developing iNTS disease compared with HIV-uninfected individuals ranged from 3.2 to 48.2. The strong association between HIV infection and iNTS disease in Africa was described in 1990 when Gilks et al reported an OR of 48.2 (confidence interval 13–176) in Nairobi, Kenya [81].

Malnutrition had a positive association with iNTS disease in five studies (Table 2), with the earliest study published in 2005. The OR for iNTS disease occurring in children with malnutrition compared with children without malnutrition ranged from 1.44 to 2.42. *Plasmodium falciparum* malaria had a positive association with iNTS disease in three African studies (Table 2) since the year 2000. The estimated OR for iNTS disease in individuals with *Plasmodium falciparum* malaria compared with those without *Plasmodium falciparum* malaria ranged from 1.5 to 4.1. In one study, recent history of malaria was positively associated with iNTS disease[192]. Young age was associated with iNTS disease in three studies (Table 2). The OR for iNTS disease in young individual compared to older individuals ranged from 2.07 to 4.30. In one study of children 1–4 years of age, iNTS disease was reported to be more associated with age when compared to other organisms causing bacteraemia[122].

Anaemia (especially moderate and severe anaemia), was shown by five studies to be associated with iNTS disease in Africa with reported OR ranging from 1.86 to 35.6. Rural settlement compared to urban was found by one study to be associated with iNTS disease [192]. The OR of iNTS disease occurring in a rural settlement compared to an urban settlement was 2.23

### Case Fatality Rate (CFR) of iNTS disease in Africa

We were able to extract CFR data for iNTS disease in Africa from twenty four studies (Table 3), describing a total of 548 deaths among 2656 cases. The overall CFR was 20.6%. and ranged from 0% in individuals greater than or equal to 5 years old in Kenya [75] to 72.7% in another Kenyan study involving only HIV-infected patients [81]. The average CFR, derived from 8 studies [3,67,89,93,152,160,172,188] conducted among low risk populations (not HIV-infected, anaemic, malnourished, or having malaria), and with >90 iNTS cases isolated, is 19% (276 fatalities from 1427 cases).

**Table 3:** CFR of iNTS disease from studies in Africa (1966 to 2014)

Author	Ref no.	Location; Study dates	CFR %	Setting	Subjects	Study entry criteria	# iNTS cases	# Deaths	Comments
Bachou et al 2006	[41]	Kampala, Uganda; Sep 2003 to Dec 2004	**23.3**	rural, hospital	Inpatients < 5 years old	Severe malnutrition	30	7	HIV-infected subjects
Bassat et al 2009	[45]	Southern Mozambique, Mozambique; June 2003 to May 2007	**25.0**	rural, hospital	Inpatients ≤ 5 years old	severe malaria	8	2	
Berkozwit et al 1984	[50]	Soweto, South Africa; January to Dec 1982	**19.6**	urban, hospital	Inpatients < 11 years old	All admissions	56	11	
Blomberg et al 2007	[51]	Dar es Salam, Tanzania; August 2001 to August 2002	**34.4**	Hospital	Inpatients ≤ 7 years old	clinical septicaemia	32	11	21.6% malaria prevalence amongst participants
Bouallegue-Godet et al 2005	[52]	Sousse, Tunisia; July 2002	**66.7**	urban, hospital	inpatients, neonates	ND	3	2	Neonates
Brent et al 2006	[3]	Kilifi, Kenya; August 1998 to July 2002	**21.1**	rural, hospital	Inpatients < 13 years old	*Salmonella* blood culture positive	166	35	
Dube et al 1983	[65]	Lusaka, Zambia; March to August 1980	**20.0**	hospital	Inpatients ≤ 2 years old	Clinical infection	10	2	Mostly neonates
Enwere et al, 2006	[67]	Basse & Bassang, Gambia; August 2000 to April 2004	**4.3**	rural, hospital	vaccine trial subjects, 2 to 29 months	Fever	92	4	Death within 28 day period
Feikin et al 2012	[75]	Asembo, Kenya; March 2007 to February 2010	**0.0**	rural, hospital	in & outpatients ≥ 5 years old	respiratory illness	41	0	37.9% HIV prevalence
Gilks et al 1990	[81]	Nairobi, Kenya; Nov 1988 to May 1989	**72.7**	urban, hospital	All ages	Inpatients	11	8	10 were HIV-infected with bacteraemia on admission
Gordon et al 2002	[82]	Blantyre, Malawi;	**47.0**	rural & urban, hospital	Inpatients ≥ 18 years old	Not specified	100	47	99% HIV prevalence
Gordon et al 2001	[84]	Blantyre, Malawi; Dec 1997 to Nov 1998	**32.9**	urban, hospital	Inpatients ≥14 years old	Fever	164	54	92% HIV prevalence amongst 25 NTS cases
Graham et al 2000	[86]	Blantyre, Malawi; February 1996 to April 1998	**24.1**	hospital	Inpatients ≤ 14 years old	Bacteraemia	241	58	HIV infected subjects, 31% malaria parasitaemia prevalence
Grant et al 1998	[88]	Abidjan, Cote d'Ivoire; Dec 1995 to March 1996	**50.0**	urban, hospital	Inpatients ≥ 18 years old	respiratory illness	14	7	HIV infected subjects, CFR includes 3 E. Coli cases
Green et al 1993	[89]	Western Zaire, D.R.C; January 1982 to Dec. 1986	**22.7**	rural, hospital	in & outpatients ≤ 5 years old	Clinical *Salmonella*	172	39	
Hadfield TL et al 1985	[93]	Zorzor, Liberia; October 1980 to August 1982	**27.8**	hospital	Inpatients ≤ 16 years old	S. Enteritidis blood culture positive	97	27	
Mandomando et al 2009	[122]	Manhiça, Mozambique; May 2001 to April 2006	**12.2**	rural, hospital	Inpatients < 10 years old	Fever	344	42	NTS associated with severe malnutrition, anaemia and age
Phoba et al 2014	[152]	Equateur, Democratic Republic of Congo; Nov 2011 to May 2012	**12.0**	hospital			83	10	69.7% has *P*. *falciparum* infection
Pithie et al 1993	[154]	Harare, Zimbabwe;	**40.7**	hospital	All ages	Bacteraemia	27	11	50% HIV prevalence
Sigauque et al 2009	[160]	Manhiça, Mozambique; May 2001 to April 2006	**12.2**	rural, hospital	Inpatients < 10 years old	All admissions	344	42	
Sow S.O. 2011	[188]	Bamako, Mali; July 2002 to June 2010	**20.0**	hospital	Inpatients ≤ 10 years old	Fever	434	87	
Tabu et al 2012	[164]	Asembo, Kenya; October 2006 to September 2009	**11.7**	rural, hospital	≤ 50 years old	Fever, respiratory illness, medical admissions	60	7	
Vaagland et al 2004	[165]	Northern Tanzania, Tanzania; July to August 2000	**60.0**	rural, hospital	Inpatients ≤ 6 years old	Clinical septicaemia	5	3	
Walsh et al, 2000	[172]	Blantyre, Malawi; Sept. 1996 to August 1997	**26.2**	rural & urban, hospital	Inpatients < 15 years old	Fever	122	32	median age for iNTS cases—15.5 months
		**Total**					**2656**	**548**	

## Discussion

Our review seeks to comprehensively document reports of iNTS disease in Africa: the number and location of the reported cases, and where available, the age of the subjects, risk factors, case fatality rates and incidence. As a result, we have included many more studies (199) than a recent review by Ao et al [204] that specifically only included reports of incidence. In their database, in addition to reports from other countries they list 10 reports from 6 African countries. All 10 reports from the Ao et al study are included in the larger set of 14 reports describing incidence from 8 African countries we include in Table 1. Not surprisingly we find a similarly high incidence of iNTS disease in sub-Saharan Africa. Ao et al estimated the overall incidence of iNTS disease in Africa at 227 cases [range 152–341] per 100,000 population with 1.9 [range 1.3–2.9] million cases annually [204]. We sought to provide a comprehensive review of the published reports of NTS bacteraemia in the peer reviewed literature and found a substantial number of reports with the earliest in 1966, interestingly about the same time as genetic studies suggest the evolution of the highly African-specific highly invasive NTS genotypes [205,206].

There are limits on the comprehensiveness of our study. In particular, many of the early and non-English reports in the initial screening stage were only available to us on line as abstracts, and in some cases, just the title with MESH headings and some of these may have contained details e.g. the use of blood cultures to determine *Salmonella* bacteraemia in the text but not in the abstract that resulted in these articles not being ordered and reviewed in full. Despite these limitations, this survey highlights important findings.

As shown in Fig 2, no reports were found in some countries in West Africa and in South West Africa. In view of the reported cases from neighbouring countries it seems unlikely that iNTS disease does not occur in these counties, but this highlights that gaps in the published literature almost certainly exist, e.g. due to lack of infrastructure in these countries to undertake the blood culturing required to obtain a definitive diagnosis, or the lack of researchers interested in publishing reports. Some of these gaps in the database may be addressed by examining health service records, but this is beyond the scope of the article.

As shown in Table 1 in the papers we surveyed, the estimated incidence of iNTS disease ranged from 1.4 cases per 100,000 population per year over all age groups in South Africa to a yearly cumulative incidence of 2,520 per 100,000 among <5 years old children in Ghana. However, we caution against trying to over-interpret these incidence data or the range observed–a striking observation is the lack of consistency in the age groups reported, the inclusion criteria that may or may not select for populations with specific risk factors (e.g. HIV, sickle cell anaemia) and in the way in which the population denominator is determined for these largely facility-based studies.

On the basis of the whole database of reported bacteraemia, we confirm the earlier findings that NTS accounts for a large proportion of the bacteria responsible for community acquired blood stream infections and is a substantial cause of morbidity and mortality with the serovars Typhimurium and Enteritidis responsible for 91% of NTS bacteraemia in Africa in the 69% of cases where the serotype was determined.

A major knowledge gap exists in the incidence of iNTS disease in Africa owing to the paucity of reports from population-based surveillance of *Salmonella* in Africa. This is due to poor surveillance systems for infectious diseases in most parts of Africa. The majority of the studies on iNTS disease in Africa are hospital-based which can only provide an estimate of the minimal incidence of disease in the community, because not all febrile cases report to hospital. Denominators necessary for the detailed estimation of iNTS disease incidence in the reported publications are generally not available. This also makes it difficult to estimate the true population at risk of disease. Population-based incidence studies are best suited to estimate the incidence of an infectious disease in Africa. This can be done by either adjusting the data obtained from hospital-based study with data obtained from simultaneous health-seeking surveys carried out in the same community (for example, as has been carried out by the Typhoid Surveillance in Africa Program (TSAP) and Severe Typhoid in Africa programme [207] or designing a study with a method that will allow for identification of all the cases of the disease within the target community.

Epidemiological data on iNTS disease were available from publications in peer-reviewed journals from 33 of the 54 countries in Africa (Fig 2). Unavailable data from the remaining 21 African countries might be due to a lack of studies in these countries, or a true low burden of iNTS disease. These 21 countries are not involved in TSAP. It would therefore be valuable to conduct a thorough search of grey literature in the affected countries including reviewing existing databases of blood cultures carried out in major hospitals in various locations across the affected countries in the last five to ten years. Such an analysis would facilitate a better understanding of the relative importance of NTS as a cause of community acquired blood stream infection in Africa. New prospective hospital-based blood culture studies in these countries would be even more useful.

The average case fatality rate of community acquired severe infections is 20.6% but with a wide range from 0 to 72%. Therefore, the development of an effective vaccine against NTS for Africa would be an important intervention to help reduce the burden of disease and deaths due to NTS. The exact mechanisms of transmission of iNTS disease are currently unclear and there are no rapid diagnostic tests available for its detection. The development of such diagnostics would greatly facilitate the study and management of iNTS disease in Africa.

## Supporting Information

S1 Checklist Prisma Checklist. (DOCX)

S1 Fig Map of Africa showing regions and countries (TIF)

S1 Table Fields used to define the Microsoft Excel data extraction template (XLSX)

S2 Table Studies identified with duplicate reports or possible overlapping cases of iNTS disease (DOCX)

S3 Table List of regions and countries with numbers of iNTS disease cases reported from Africa (till 2014) (DOCX)

S4 Table Reports obtained according to study site setting (DOCX)

S5 Table Causes of community acquired bacteraemia and NTS proportion in Africa (1966–2014) (DOCX)
