# Supplementary figures and images for: A Systematic Review of the Incidence, Risk Factors and Case Fatality Rates of Invasive Nontyphoidal Salmonella (iNTS) Disease in Africa (1966 to 2014)
Source: PLoS Negl Trop Dis. 2017 Jan 5;11(1):e0005118. doi: 10.1371/journal.pntd.0005118 (PMC5215826; doi:10.1371/journal.pntd.0005118)

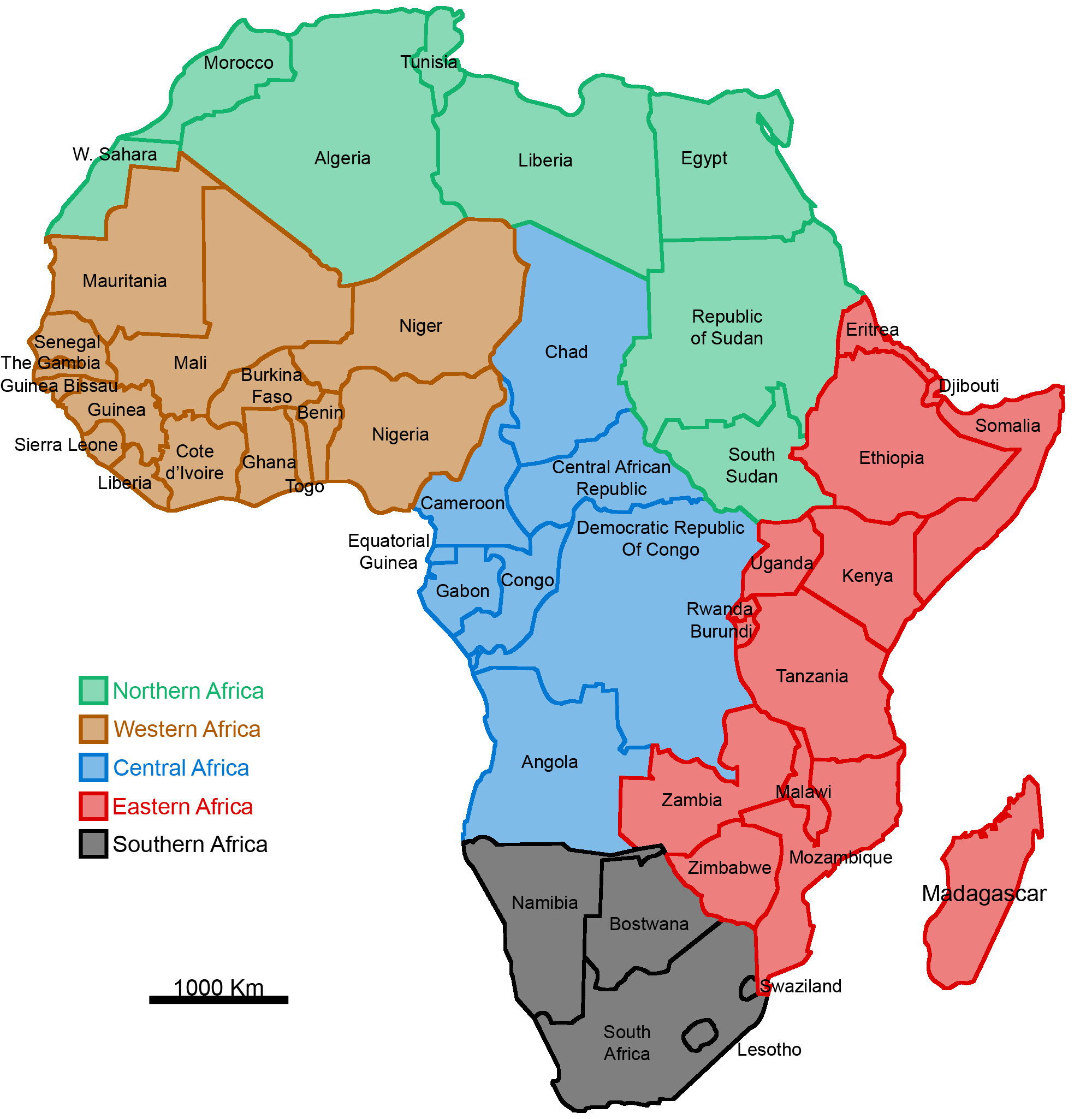

Supplement: S1 Fig — (TIF) [file pntd.0005118.s002.tif]
