# Supplementary material for: A Systematic Review of the Incidence, Risk Factors and Case Fatality Rates of Invasive Nontyphoidal Salmonella (iNTS) Disease in Africa (1966 to 2014)
Source: PLoS Negl Trop Dis. 2017 Jan 5;11(1):e0005118. doi: 10.1371/journal.pntd.0005118 (PMC5215826; doi:10.1371/journal.pntd.0005118)
Supplement: S2 Table — (DOCX) [file pntd.0005118.s004.docx]

S2_Table: Studies identified with duplicate reports or possible overlapping cases of iNTS

| **Studies excluded from the quantitative analysis because of duplication** | | | | | |
| --- | --- | --- | --- | --- | --- |
| **S/N** | **Study** | **Ref no** | **Details included in** | **Ref no** | **Comments** |
| 1 | Brent et al, 2006 | 3 | Berkley et al, 2005 | 2 | Kenya |
| 2 | Feasey et al, 2010 | 11 | Gordon et al, 2008 | 83 | Duplicated cohort from Malawi |
| 3 | Maltha et al, 2014 | 121 | Maltha et al, 2014 | 120 | Burkina Faso |
| 4 | Mandomando et al, 2009 | 122 | Sigauque et al, 2009 | 160 | Mozambique |
| 5 | Ley et al, 2014 | 181 | Lunguya et al, 2013 | 117 | DRC |
| 6 | Ley et al, 2010 | 114 | Ley et al, 2011 | 115 | Tanzania; 114 is very likely a subset of 115 |
| 7 | Biggs et al, 2014 | 192 | Nadjm et al, 2010 | 198 | Tanzania |
| 8 | Nadjm et al, 2010 | 198 | Mtove et al, 2011 | 129 | Tanzania |
| 9 | Mtove et al, 2010 | 197 | Mtove et al, 2011 | 129 | Tanzania |
| 10 | Dakoury-Dogbo et al, 2001 | 193 | Anglaret et al, 2002 | 29 | Cote d'Ivoire |
| 11 | Schwarz et al, 2010 | 199 | Nielsen et al, 2012 | 137 | Ghana |
| 12 | Mandomando et al, 2010 | 196 | Sigauque et al, 2009 | 160 | Mozambique |
| 13 | Enwere et al, 2007 | 194 | Enwere et al, 2006 | 67 | Gambia; 194 is very likely a subset of 67 |
| 14 | Jacob et al, 2013 | 195 | Jacob et al, 2012 | 98 | Uganda; 195 is very likely a subset of 98 |
| **Studies included in quantitative analysis, with possible overlap of iNTS reports** | | | | | |
| **S/N** | **Study** | **Art no** | **Likely overlap with** | **Art no** | **Comments** |
| 1 | Wiktor et al, 1999 | 173 | Anglaret et al, 2002 | 29 | Cote d'Ivoire |
| 2 | Lunguya et al, 2013 | 117 | Phoba et al, 2012 | 153 | DRC |
| 3 | Arthur et al, 2001 | 35 | Gilks et al, 1990 | 81 | Kenya; 81 is likely a subset of 35 |
| 4 | Feikin et al, 2012 | 75 | Tabu et al, 2012 | 163 | Kenya |
| 5 | Kariuki et al, 2006 | 104 | Kariuki et al, 2005 | 103 | Kenya |
| 6 | Williams et al, 2009 | 174 | Berkley et al, 2005 | 2 | Kenya |
| 7 | Gordon et al, 2001 | 84 | Gordon et al, 2008 | 83 | Kenya |
| 8 | Archibald et al, 2003 | 30 | Archibald et al, 2003 | 33 | Kenya |
| 9 | Nhampossa et al, 2013 | 136 | Sigauque et al, 2009 | 160 | Mozambique |
| 10 | Lepage et al, 1984 | 109 | Lepage et al, 1990 | 110 | Rwanda |
| 11 | Enwere et al, 2006 | 67 | Ikumapayi et al, 2007 | 97 | Gambia |
| 12 | Jacob et al, 2009 | 99 | Jacob et al, 2012 | 98 | Uganda |
