# Supplementary material for: A Systematic Review of the Incidence, Risk Factors and Case Fatality Rates of Invasive Nontyphoidal Salmonella (iNTS) Disease in Africa (1966 to 2014)
Source: PLoS Negl Trop Dis. 2017 Jan 5;11(1):e0005118. doi: 10.1371/journal.pntd.0005118 (PMC5215826; doi:10.1371/journal.pntd.0005118)
Supplement: S3 Table — (DOCX) [file pntd.0005118.s005.docx]

S3_Table: List of countries with frequency of iNTS reported from Africa (till 2014)

| **Region/Countries** | **Publication frequency** | **Total iNTS isolates** | **S. Typhimurium** | **S. Enteritidis** |
| --- | --- | --- | --- | --- |
| **Eastern** | **94** | **11211** | **6580** | **1820** |
| Burundi | 1 | 54 | 44 | 10 |
| Comoros | 1 | 23 |  | 23 |
| Ethiopia | 6 | 43 |  |  |
| Kenya | 28 | 2976 | 1169 | 431 |
| Malawi | 24 | 6057 | 4237 | 1150 |
| Mauritius | 2 | 35 | 4 |  |
| Mozambique | 4 | 513 | 290 | 102 |
| Rwanda | 3 | 344 | 321 | 23 |
| Tanzania | 12 | 347 | 16 | 25 |
| Uganda | 10 | 767 | 495 | 46 |
| Zambia | 1 | 10 | 4 |  |
| Zimbabwe | 2 | 42 |  | 10 |
| **Middle** | **17** | **1180** | **527** | **354** |
| Cameroon | 1 | 5 |  |  |
| Central African Republic | 2 | 34 | 19 | 14 |
| Democratic Republic of the Congo | 11 | 1003 | 470 | 314 |
| Gabon | 3 | 138 | 38 | 26 |
| **Northern** | **12** | **1163** | **42** | **15** |
| Algeria | 2 | 1023 | 37 |  |
| Egypt | 1 | 1 |  |  |
| Morocco | 2 | 36 |  | 11 |
| Sudan | 2 | 3 |  |  |
| Tunisia | 5 | 100 | 5 | 4 |
| **Southern** | **7** | **2819** | **1565** | **134** |
| South Africa | 7 | 2819 | 1565 | 134 |
| **Western** | **46** | **2558** | **370** | **478** |
| Burkina Faso | 3 | 198 |  |  |
| Cote d'Ivoire | 9 | 371 | 6 | 37 |
| Ghana | 8 | 345 | 22 | 93 |
| Guinea-Bissau | 1 | 5 |  |  |
| Liberia | 1 | 109 |  | 109 |
| Mali | 3 | 788 | 220 | 32 |
| Niger | 1 | 14 |  |  |
| Nigeria | 9 | 180 | 11 | 24 |
| Senegal | 4 | 243 | 77 | 101 |
| The Gambia | 6 | 245 | 15 | 50 |
| Togo | 1 | 60 | 19 | 32 |
| **Grand Total** | **176** | **18931** | **9084** | **2801** |
