# Supplementary material for: A Systematic Review of the Incidence, Risk Factors and Case Fatality Rates of Invasive Nontyphoidal Salmonella (iNTS) Disease in Africa (1966 to 2014)
Source: PLoS Negl Trop Dis. 2017 Jan 5;11(1):e0005118. doi: 10.1371/journal.pntd.0005118 (PMC5215826; doi:10.1371/journal.pntd.0005118)
Supplement: S4 Table — (DOCX) [file pntd.0005118.s006.docx]

S4_Table: Reports obtained according to study site setting

| Type of setting | Reports (freq) | iNTS (freq) |
| --- | --- | --- |
| rural | 44 | 3146 |
| rural & urban | 12 | 2103 |
| urban | 57 | 7521 |
| Not Specified | 63 | 6161 |
| Grand Total | **176** | **18931** |
