# Supplementary material for: A Systematic Review of the Incidence, Risk Factors and Case Fatality Rates of Invasive Nontyphoidal Salmonella (iNTS) Disease in Africa (1966 to 2014)
Source: PLoS Negl Trop Dis. 2017 Jan 5;11(1):e0005118. doi: 10.1371/journal.pntd.0005118 (PMC5215826; doi:10.1371/journal.pntd.0005118)
Supplement: S5 Table — (DOCX) [file pntd.0005118.s007.docx]

**S5_Table: Causes of community acquired bacteremia and iNTS proportion in Africa (1966-2014)**

| **Region/ Country** | **Ref no** | **Total BCs** | **Bacteremia** | **iNTS prop** | **S. Typhi** | **iNTS** | **Hib** | **E. coli** | **Neisseria** | **Other Gram**  **-ve** | **S. pneumonia** | **S. aureus** | **Other Staph** | **Other Gram +ve** |
| --- | --- | --- | --- | --- | --- | --- | --- | --- | --- | --- | --- | --- | --- | --- |
| **Eastern** | **36** | **103896** | **14726** | **0.27** | **392** | **4319** | **332** | **797** | **32** | **1313** | **1361** | **1211** | **236** | **1051** |
| Ethiopia | 27, 37 | 1582 | 314 | 0.09 | 9 | 16 |  | 21 |  | 76 | 6 | 68 | 103 | 15 |
| Kenya | 23,46,64,102,107,142 | 21365 | 3031 | 0.28 | 130 | 285 | 101 | 222 | 15 | 424 | 616 | 586 | 133 | 293 |
| Malawi | 30,31,32,33, 48,73,83,84, 92,125,151, 171,172 | 50080 | 8585 | 0.39 | 91 | 3272 | 22 | 261 | 1 | 516 | 177 | 263 |  | 556 |
| Mozambique | 160 | 19896 | 1550 | 0.26 | 3 | 397 | 113 | 157 | 16 | 157 | 388 | 189 |  | 126 |
| Rwanda | 111 | 900 | 112 | 0.32 | 47 | 36 | 3 | 1 |  | 1 | 14 | 9 |  | 1 |
| Tanzania | 51,60,62,119,124,129,  130,132,179 | 9072 | 1018 | 0.17 | 112 | 282 | 93 | 126 | 0 | 135 | 128 | 58 | 0 | 60 |
| Uganda | 39,98,99,163 | 1001 | 116 | 0.19 |  | 31 |  | 9 |  | 4 | 32 | 38 |  |  |
| **Middle** | **5** | **2956** | **681** | **0.38** | **44** | **283** | **2** | **29** | **1** | **190** | **19** | **12** | **5** | **11** |
| C.A.R | 105 | 131 | 49 | 0.45 | 2 | 22 |  | 2 |  | 2 | 6 |  | 3 |  |
| D.R.C | 42,43,152,167 | 2825 | 632 | 0.36 | 42 | 261 | 2 | 27 | 1 | 188 | 13 | 12 | 2 | 11 |
| **Southern** | **3** | **0** | **12891** | **0.08** | **911** | **375** | **370** | **1671** | **139** | **3919** | **1229** | **1933** | **31** | **2487** |
| South Africa | 50,59,166 | 0 | 12891 | 0.08 | 911 | 375 | 370 | 1671 | 139 | 3919 | 1229 | 1933 | 31 | 2487 |
| **Western** | **12** | **7782** | **1509** | **0.18** | **295** | **269** | **49** | **49** | **3** | **180** | **111** | **199** | **1** | **32** |
| Burkina Faso | 120,145 | 842 | 145 | 0.34 | 31 | 49 | 1 | 12 | 2 | 18 | 12 | 13 |  | 7 |
| Cote d'Ivoire | 66,168 | 2108 | 412 | 0.15 | 99 | 59 |  | 6 |  | 14 | 4 | 40 |  |  |
| Ghana | 44,90 | 800 | 182 | 0.25 | 59 | 48 |  | 1 |  | 20 | 1 | 40 |  |  |
| Guinea-Bissau | 176 | 372 | 46 | 0.11 | 3 | 5 | 0 | 2 | 0 | 5 | 4 | 26 | 0 | 3 |
| Nigeria | 25,138 | 2789 | 251 | 0.08 | 46 | 19 | 4 | 19 |  | 72 | 10 | 63 | 1 | 14 |
| Gambia | 95,118,139 | 871 | 473 | 0.15 | 57 | 89 | 44 | 9 | 1 | 51 | 80 | 17 |  | 8 |
| **Grand Total** | **56** | **114634** | **29807** | **0.25** | **1642** | **5246** | **753** | **2546** | **175** | **5602** | **2720** | **3355** | **273** | **3581** |
